# A Manual of Chemistry

**Published:** 1893-06

**Authors:** 


*A Manual of Chemistry*. By ARTHUR P. LUFF, M.D., B.Sc. Pp. xvi., 525. London: CASSELL & COMPANY, LIMITED. 1892.

This book is intended to serve as a medical students' guide to the study of chemical science. It is one of the red-cover series, most of which have already proved themselves to be good friends to the medical student: it appears to supply a real want, and will assist the student in obtaining a sound knowledge of what is essential to every medical practitioner. As a rule, chemistry is a thing to be tolerated; the student has not that love for it which it deserves. This manual will facilitate its study, and will lay a better foundation for physiology and pathology than is generally laid.

